# 

**Published:** 1886-04

**Authors:** 


### TO THE MEMBERS OF THE MEDICAL ASSOCIATION OF GEORGIA.

The next meeting of the Medical Association of Georgia will be held in Augusta, April 21, 22 and 23.

The railroad rates to members attending this meeting have been fixed at one and one-third fares for the round trip. Members must pay full fare going, and will be furnished with a certificate by the Secretary, which will be signed by them in the presence of agent at the starting point, which, when signed by the Secretary at Augusta, will entitle them to return at the rate of one cent per mile.

No reduced return ticket will be issued unless the party can show a certificate signed by the agent from whom he purchased the going ticket and signed by the Secretary of the Association at Augusta.

The hotels will charge members from $1.00 to $2.50 per day.

I am informed by the Chairman of the Committee of Arrangements, Dr. E. C. Goodrich, that the outlook for an extra large attendance is promising. Every arrangement has been made to entertain the Asssociation in the best style.

Every member who can should be present.

JAMES A. GRAY, M. D., *Secretary.*

